# Assessing strength of evidence for regulatory decision making in licensing: What proof do we need for observational studies of effectiveness?

**DOI:** 10.1002/pds.5005

**Published:** 2020-04-16

**Authors:** Jim Slattery, Xavier Kurz

**Affiliations:** ^1^ European Medicines Agency Amsterdam The Netherlands

## Abstract

Before a medicine can be recommended for a marketing authorization research must be provided to regulators that convincingly supports the benefit‐risk of the product in the claimed indication. The established criteria for such research are usually expressed in terms of evidence from randomized controlled trials (RCT). If studies in real‐world data (RWD) are to be accepted as all or part of the package of evidence, it is necessary to understand the relationship between information from studies of RWD and that from RCTs. The aim of this review is to consider how the strength of such evidence can be quantified in a manner that relates to the decision‐making process, what research is currently available to further this understanding and what additional information will be required.

**Key points**

- Availability of large quantities of observational data from clinical practice and health insurance systems has prompted suggestions of a potential role in supporting regulatory assessment of drug effectiveness.
- In order to protect public health, regulators must understand the reliability of the evidence underlying their decisions.
- Analyses of observational data are prone to biases that necessitate empirical evaluation.
- Large‐scale experiments to measure errors in observational studies are already under way and will inform decisions on how the results of such studies can be used by regulators.
- Additional work will be required to ensure that the design of future studies conform to validated standards and that their conduct can be verified by regulators.

## 1 STRENGTH OF EVIDENCE: WHAT DO WE MEAN?

A responsibility of drug regulators is to check that evidence supports a favorable benefit‐risk profile throughout the product lifecycle. An early step in this process is to ensure that ineffective medicines never enter the market. The way that they do this is through evaluation of the research evidence supplied to them by the company wishing to market the product. This raises the question of what type of evidence is sufficient to allow the regulator to be confident of its decision. Regulators might like to require overwhelmingly convincing evidence but, as in all decision processes, there is a balance to be considered. If we demand extremely strong evidence the data will be difficult and very time‐consuming to collect and, consequently, the entry of good products into clinical practice may be delayed and their cost increased. Conversely, if we ask for too little evidence products with little clinical effect will slip through the net. These considerations highlight the fact that we need to be able to clearly define what we mean by “strength of evidence” and specify how it can be evaluated with respect to any chosen type of research.

Most of us have an idea of what we mean by strength of evidence. Strong evidence is that which predisposes us to believe a fact firmly while weaker evidence leaves us with more doubt. However, such subjective notions are unsatisfactory for drug licensing: a formal decision‐making process that must apply equitable and verifiable standards across manufacturers and maintain the standards over time. One approach is to specify precisely the type and quantity of research that must be presented in support of a marketing authorization application and the nature of the results of that research that we would consider to support the conclusion that the drug is a useful medicine. Up till now this approach has proved to work for most drug licensing applications and, following much debate in the 1980s and 90s, the requirements have usually been phrased in terms of randomized controlled trials (RCT) which are formal experiments with dedicated data collection processes. However, other forms of study exist and some attention has lately been given to studies using observational data collected for purposes other than research, often in clinical practice or as part of the reimbursement process. These data are a subset of real‐world data (RWD). For regulatory definitions of RWD and a useful discussion of the strengths and challenges it is worth reading Beaulieu‐Jones.^1^ Of course, the need to consider other types of study complicates the discussion of strength of evidence. If we allow a wider range of study methodology the specification of exactly what types of study and results will be acceptable becomes extremely challenging. However, the general aim remains that, no matter what form of study is used, we would like the decision process regarding approving the product to be equally reliable. To achieve this, we need to understand what the traditional specification of strength of evidence in terms of RCTs implies about the properties of our decision‐making process and how a similar understanding can be reached for other forms of research.

Fortunately, at the highest level, the characterization of strength of evidence is relatively simple. As discussed, practical decision processes are never perfect and their imperfections result in occasional incorrect decisions. In the case of drug authorization process, a proportion of drugs that do not have the intended clinical effect will be granted licenses—the false positive rate (FPR)—and a proportion of products that do work will be refused—the false negative rate (FNR). Thus, the problem of maintaining the same strength of evidence reduces to keeping the same FPR and FNR no matter what form of evidence is used. More details of these concepts can be found in the literature on statistical decision theory.^2^, ^3^

## 2 STRENGTH OF EVIDENCE UNDER CURRENT REGULATORY GUIDELINES

For a substantial majority of marketing applications pivotal RCTs are expected.^4^ For an RCT with complete follow‐up and adequate concealment of the treatment allocation, the FPR, referred to as the type 1 error rate, and FNR at a chosen effect size, the type 2 error rate, are parameters that are built into the design of the study.^5^ Because there is no systematic bias in the allocation of the treatments it is possible to calculate the distribution of differences in outcome between the treatment groups under the assumption that no treatment effect exists. Hence the probability of a false positive can be exactly calculated for any trial and, by increasing the sample size, it can be reduced to any value we choose while keeping the FNR steady. Similarly, for any supposed treatment effect to be detected, we can calculate and control the FNR. Thus, strength of evidence can be controlled by the experimenter.

Of course, studies using observational data are already occasionally accepted in clinical areas where RCTs are difficult. In this respect, Banzi^6^ gives a critical review of evidence accepted in support of conditional approvals, and Pontes^7^ develops a classification of clinical scenarios that may help to standardize submissions. Hatswell^8^ reviews approvals without RCT evidence by EMA and FDA over 1999 to 2014 and recommends guidelines to describe an acceptable data package for regulators. This article also provides rough estimates of potential delay from waiting for stronger evidence (mean = 21.5 m). It is noted that perceptions of what appears to be adequate evidence are inconsistent and it is this point that motivates our current discussion.

## 3 GENERALIZING TO RESEARCH OTHER THAN RCTS

Although it is customary for more than one study to be submitted in support of any application to market a product, it is worth thinking first about single studies. Unfortunately, the error rates are not easily calculable in studies using observational data. Moreover, in contrast with RCTs, there is no way to reduce them to any chosen value. One reason for this difficulty is that patients who receive a treatment tend to differ from those who do not. Often this is because treatments in clinical practice are given preferentially to patients who appear to need them, and these patients are systematically different from untreated patients. When we see differences in outcome between treated patients and untreated patients, we ask whether the differences are due to the treatment or to natural differences between the patient groups. Of course, this has been known for many years and the fact that such bias complicates all observations in “real life” was exactly the reason that randomization has become the preferred approach to scientific research where the allocation of interventions can be controlled by the researcher.

Although it is difficult to determine the strength of evidence from observational studies, there are good reasons to use such data in many areas of research and effort has been invested in developing methods to control the bias in study results. The existence of such methods and the reliance placed on this research in some important and difficult areas such as criminal justice, education, social work, road safety, environmental policy and not least, in drug safety, raises the question of whether this type of research could play a more important role in deciding which patients can be treated with a medical product for a given disease.

## 4 EMPIRICAL APPROACHES TO STRENGTH OF EVIDENCE

Some observational researchers acknowledge the doubt surrounding the question of strength of evidence and have attempted to address it using scientific methods. As a prelude, we should consider what has been observed with respect to past studies.

In 2005, John Ioannidis published an essay called *Why most published research findings are false*.^9^ He attributes the problems to a number of causes; preferential publication of positive results, multiple testing, intentional, or unintentional selection of methods that give a preferred result, but he also notes that research finding may just be accurate measures of the prevailing bias. He also observes that positive research results are commoner when there are financial or other interests involved. Of course, many of these also affect experimental research and, under controlled conditions, only two are significant challenges in drug development: the bias intrinsic to the data collection procedure and the potential for a researcher to inappropriately influence the results of an analysis. Thus, it is reasonable to conclude that Ioannidis' findings represent a more pessimistic picture than we would expect when appropriate regulation of research is exercised.

Two questions arise in relation to use of new methods in drug development:

1. Can the methods when used according to best practice provide the required strength of evidence?
2. Can regulatory guidelines be designed and enforced in a manner that assures adherence to best practice?

The first of these questions is currently being studied by a number of researchers and we have selected two large projects, in particular, as important examples of ongoing effort.

Following on from 21st Century Cures Act the U.S. FDA^10^ committed to investigate the possible role of real‐world evidence in drug development and, in particular, in assessing new indications for established products. Part of this investigation involves a project to replicate the results of 30 RCTs using an unrandomized cohort study in claims databases—a form of observational data arising from health insurance systems.^11^, ^12^ Most of these RCTs showed positive effects and thus the FDA has chosen in this first phase to concentrate on estimating the FNR, in addition to the possible systematic errors in the studies that returned positive results. The replicating cohort studies make every effort to mirror the types of patients and outcome measures used in the original trials but the actual exposure of the patients to the medicines may be less regimented than in a formal study. The complete results of this investigation will be available in March 2020. A later stage of the project will include replication of seven ongoing trials for which there will be no concern that knowledge of the trial results could bias the cohort studies.

The FPR is a matter of more immediate concern to regulators than the FNR. Licensing ineffective medicines could do substantial damage to patients as it might delay receipt of effective treatment or simply prolong suffering while unnecessarily exposing patients to potentially harmful effects. A major investigation of false positives in observation studies under highly standardized conditions—avoiding many of the problems discussed by Ioannidis—has been carried out using the ATLAS system^13^ which allows very large numbers of study questions to be addressed in an automated fashion. Schuemie et al identified 15 medical interventions and 52 negative controls—outcomes which they were confident could not be related to the intervention—and ran cohort studies with high‐dimensional propensity score adjustment. This particular report was restricted to antidepressant therapies and was run in three different databases. The results showed that, of those results that proved feasible to obtain, using a nominal rate of 5%, about 15% were false positives. In other words, about three times as many as might have been hoped for but nowhere near as many as in the uncontrolled research setting examined by Ioannidis. For completeness, it should be noted that Schuemie used his results to adjust the formal criteria for statistical significance to calibrate the FPR back to 5% with the penalty of an increase in FNR. In evaluation of the acceptability of these error rates it is worth bearing in mind that one can be traded off against the other. Hence, in selecting study methods for drug development, regulators could specify the FPR that is considered acceptable and companies would then decide if the FNR represented an acceptable risk.

Are these results promising or not as regards reaching the desired strength of evidence for regulatory decision making? The answer is not straightforward. Schuemie's study suggests that some appreciable control of the FPR is possible. However, when differences between the three data sources and between outcomes are investigated significant systematic variation is detectable.^1^ This means that no across‐the‐board statement can be made about the FPR in observational studies. Moreover, this was in a single clinical area and hence further variation may emerge with examination of other disease areas. Also, there was no variation in investigator choices of methodology between research questions because all questions were addressed in an identical fashion by a single computer program.

As noted above, we also need to understand how the error rates from different studies can be combined into single values. The arguments are quite complex but, once again, theoretical methods are used for multiple RCTs that become more complex for observational studies because different studies of the same drug and outcome will tend to exhibit similar biases. These biases cannot be theoretically predicted but must be estimated from data. The recent development of very large and detailed repositories of clinical data make this a realistic, if not simple, proposition.

## 5 INFLUENCE OF STUDY QUALITY

The methods discussed above to evaluate strength of evidence from observational studies could be applied to any study design. This article cannot go into detail about the possible types of study and the many fundamental design choices that must be made when implementing them. It suffices to say that much additional research effort is devoted to selection of methods that seem likely to reduce error probabilities. A promising idea is that designs should stay as close as possible to a notional RCT. Specific cases based on this idea^14^, ^15^, ^16^, ^17^ have been published and appear to suggest that with subject matter knowledge, good data, and careful choice of model it may be possible to appreciably reduce confounding and selection.

Complex study designs pose an interesting challenge for regulators. Generalization of empirical evaluations of error rates to future studies requires that the population of studies evaluated are substantively similar to new studies and this requires standardized and verifiable principles for study design. Further work is needed to standardize any chosen approach for regulatory purposes.

## 6 ASSURING HIGH QUALITY RESEARCH

The variation in results, some of which may depend on choices made by the researcher, brings us back to the second regulatory concern. Can we ensure adherence to best practice? In addition to random assignment of treatments, formal trials frequently include a control treatment superficially indistinguishable to the test product, defined procedures for data collection and prespecified outcome measures, analysis plans, and success criteria. All these features have a role in avoiding unintentional and intentional bias in the results. The question of how and at what stage bias might enter an observational study will need to be thought through in equal detail and processes designed to ensure control of the bias.

As an example, consider that most observational studies are run on data that have already been collected. In safety studies, this is a major advantage as the hypotheses have usually arisen recently and hence the data could not have been influenced by the hypothesis and a swift answer can be obtained as no further data collection is needed. By contrast, prospective indications for a medicine are often identified at an early stage of development. Thus, questions arise about whether knowledge of these planned indications affected collection of data in patients receiving the drug. This might sound unlikely, but it is a question frequently asked when safety problems have been known for some time; could this knowledge have differentially affected the recording of the event with the specific drug in comparison to alternative treatment. Regulatory control of this issue is difficult.

Ioannidis points out that false positives in observational studies appear more common when vested interests are present. This raises the matter of how selection of outcome measures, analytic techniques, and data collection procedures may be protected from selection designed to enhance a desired result. The measures discussed above work well with RCTs but are often impossible in observational study. If data have already been collected, it is difficult to ensure that an analysis plan and outcome measures have been designed in ignorance of the data.

Questions such as these will require careful thought and currently it is probably fair to say that the scientific evaluations such as those described above are somewhat in advance of the regulatory discussions and guideline production that will be necessary to support extension of the use of observational data to licensing decisions. However, this is probably justified when we are not yet at the stage of deciding that appropriate strength of evidence can be obtained even under ideal circumstances. In order to make this decision, we need to fully characterize the levels of FPR achievable and the factors that affect it, and then to work carefully through implications of various approaches to the research on the final decision processes. It may be a long road.

## 7 CONCLUDING POINTS

The preferred strength of evidence for routine licensing applications was vigorously debated in the 1980s and 1990s. Even so, it can be an uncomfortable area for open debate as it requires recognition that no decision process can ever guarantee perfect results. Even with current licensing practice, occasional recommendations for revocation for lack of efficacy occur, for example, Xigris in the European Union^18^ and some generic methylphenidate in the United States.^19^ The current discussions over observational studies reopen and widen the scope of the argument and this is in many ways healthy. It is well known that we do occasionally accept less evidence when there appear good reasons to do so. But, with conditional or exceptional circumstances authorizations the strength of evidence is not quantified in terms of error probabilities and so it is challenging to maintain equity in regulation and also difficult to predict the net effect of such decisions on the overall health of patients. The new approaches to evaluating false decision rates in research other than RCTs may help us formalize even these areas and, possibly, widen our discussion of strength of evidence to reflect the potential of each new product to improve public health rather than applying a uniform and precautionary standards to every medicine.

## CONFLICT OF INTEREST

The authors declare no conflict of interest.

## DISCLAIMER

The views expressed in this article are those of the authors and should not be understood or quoted as being made on behalf of or reflecting the position of the European Medicines Agency or one of its committees or working parties.

## Endnote

1 Based on analysis of data made accessible by Schuemie et al but not presented here.
